# “Surviving to thriving”: a meta-ethnography of the experiences of healthcare staff caring for persons with COVID-19

**DOI:** 10.1186/s12913-021-07112-w

**Published:** 2021-10-21

**Authors:** Frank Bediako Agyei, Jonathan Bayuo, Prince Kyei Baffour, Cletus Laari

**Affiliations:** 1 Department of Nursing, Presbyterian University College, Agogo, Ghana; 2 Directorate of Surgery, Komfo Anokye Teaching Hospital, Kumasi, Ghana; 3 Department of General Nursing, School of Nursing and Midwifery, University for Development Studies, Tamale, Ghana

**Keywords:** Experiences, Healthcare professionals, Meta-ethnography

## Abstract

**Background:**

The emergence of the Coronavirus disease has heightened the experience of emotional burden among healthcare staff. To guide the development of support programmes, this review sought to aggregate and synthesise qualitative studies to establish a comparative understanding of the experiences of healthcare staff caring for persons with the disease.

**Design:**

A meta-ethnography approach was used to aggregate and synthesise primary qualitative studies. Database search was undertaken from January to November 2020. A standardised tool was used to extract data from the identified primary studies. The studies were translated into each other to formulate overarching concepts/ metaphors which formed the basis of undertaking a narrative synthesis.

**Results:**

Eight qualitative studies met the inclusion criteria. Two overarching metaphors/ concepts were formulated from the primary studies: 1) surviving to thriving in an evolving space and 2) support amid the new normal. The initial phase of entering the space of caring during the outbreak was filled with psychological chaos as healthcare staff struggled to survive within the context of an illness which was not fully understood. Gradually, healthcare staff may transition to a thriving phase characterised by resilience but still experienced heavy workload and physical/ emotional exhaustion predisposing them to burnout and compassion fatigue. Fear persisted throughout their experiences: fear of contracting the disease or infecting one’s family members/ loved ones remained a key concern among healthcare staff despite infection precaution measures. Healthcare staff who contracted the disease felt isolated with additional fears of dying alone. The sources of support were varied with a strong emphasis on peer support.

**Conclusions:**

Healthcare staff caring for persons infected with the Coronavirus disease are at risk of burnout and compassion fatigue and require ongoing mental health support commensurate to their needs. Staff who contract the disease may require additional support to navigate through the illness and recovery. Policies and concerted efforts are needed to strengthen support systems and build resilience among healthcare staff.

## Background

The emergence of the novel Coronavirus disease (COVID-19) across the globe has led to various healthcare systems becoming overwhelmed with clinicians facing significant emotional strain and physical pressure [1, 2]. Evidence from several countries suggest an increasing rate of depression, anxiety, and insomnia among healthcare workers caring for persons with COVID-19 [3–5]. These emotional/ physical pressures, if left unresolved, may lead to a higher incidence of suicide and substance abuse among healthcare workers [6–9].

Caring for critically ill persons is often associated with emotional and physical exhaustion [10–13]. The sudden occurrence of the COVID-19 pandemic, which healthcare systems were seemingly unprepared for alongside increasing mortality rates in some areas have contributed to the development of fear, worry and uncertainty [14]. These concerns are likely to increase the burden experienced by healthcare staff creating the need for ongoing support [15]. Various settings are implementing several programmes for healthcare staff but there appears to be an ever increasing need to provide ongoing evidence-based psychosocial support [16–19].

The pandemic is gradually becoming the ‘new normal’ implying that we may have to live with it for an unknown period [20–22]. In the absence of adequate/ context-specific support programmes for our healthcare providers, their well-being may be adversely affected which can affect the overall availability of human resource and even translate to poor patient care [23–25]. So far, primary studies exploring the experiences of healthcare staff caring for patients with COVID-19 are emerging which offers some insight into their lived experiences. However, to gain a broader perspective and facilitate the design of interventions (timing and nature/ components), there is a need to establish a comparative understanding of these experiences. Besides as the pandemic evolves, there is a need for robust evidence regarding clinicians’ experiences in navigating through the pandemic to understand the variations and similarities across contexts and attain a deeper breadth of the phenomenon. Such broad perspective can contribute significantly to global healthcare policy and practice particularly as it remains uncertain when the COVID-19 pandemic may end. Thus, this review sought to identify the available primary studies, aggregate, and synthesise their findings to understand the phenomenon of caring for persons diagnosed with COVID-19. The review question was “what are the experiences of healthcare professionals caring for persons with COVID-19?”

### Aim

The aim of this review was to develop a comparative understanding of the experiences of healthcare staff caring for persons with COVID-19.

## Methods

### Review design

Noblit and Hare’s approach to meta-ethnography was utilised for this review [26]. Meta-ethnography is an aggregative method of synthesis which seeks to integrate separate parts to form a whole. It involves induction and interpretation, thus resembling the primary studies it aims to synthesise [27]. In simple terms, meta-ethnography is the qualitative alternative to quantitative meta-analysis [26]. The product of a meta-ethnographic synthesis is the interpretation of the primary studies into one another to generate an in-depth/ new understanding of a phenomenon [28]. This meta-ethnography was reported according to the eMERGe reporting guidelines [29]. Additionally, the Preferred Reporting Items for Systematic Reviews and Meta-Analyses (PRISMA) flowchart [30] was used to guide the process of study selection.

### Search strategy/ study identification

A limited search in CINAHL, EMBASE and PubMed was initially undertaken which informed the development of a detailed search strategy. The full search sources included Cochrane Reviews Library, EMBASE, CINAHL, PubMed, OVID, Scopus, and Web of Science from December 2019 to November 2020. The following key words were used: “healthcare professionals” OR “healthcare practitioners” OR “healthcare staff” OR “healthcare workers” AND “COVID-19” OR “Coronavirus disease” OR “clinical respiratory illness” OR “clinical respiratory infection” OR “influenza-like illnesses to identify qualitative studies focusing on the phenomenon. The reference lists of identified articles were manually searched for potential studies.

### Study selection and screening

**Fig. 1:**
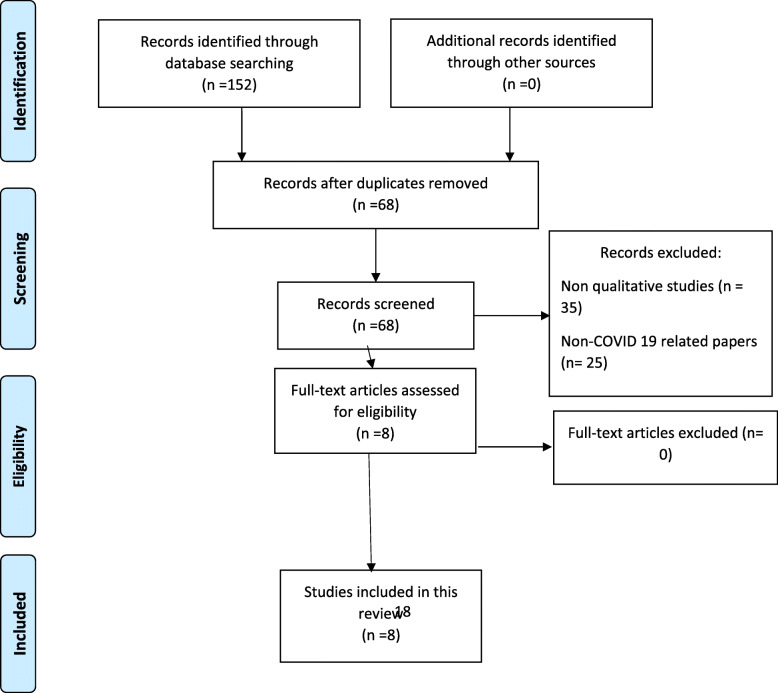
PRISMA flowchart of study selection

Upon completing the search, all identified articles were exported into Endnote X9.2 and duplicates removed. This was followed by a selection procedure to identify primary studies for inclusion in the review. The inclusion criteria were 1) primary studies exploring the experiences of healthcare professionals caring for patients with COVID-19 2) qualitative studies irrespective of the design and 3) reported in English. Preprints, grey, non-qualitative studies, commentaries, editorials, and unpublished literature were not considered for inclusion in this review. Title screening was initially carried out to ensure that the study is relevant to the review. Abstract screening was then carried out. Full texts of the studies meeting the inclusion criteria were retrieved. These studies proceeded to the critical appraisal stage before including them in the review. The results of the search are presented in the PRISMA flow diagram below as Fig. 1.

### Quality assessment/ appraisal

Studies considered for inclusion were critically appraised using the Joanna Briggs Institute (JBI) standardized critical appraisal checklist for qualitative studies. Potential studies that received an overall appraisal as ‘include’ were retained in the review (see Table 1).

**Table 1:** Quality appraisal

	Sun et al.,	Liu et al.,	Kackin et al.,	Karimi et al.,	Galehdar et al.,	Ardebili et al.,	Al Ghafri et al.,	Nyashanu et al.,
Is there congruity between the stated philosophical perspective and the research methodology?	Yes	Yes	Yes	Yes	Unclear	Unclear	Unclear	Yes
Is there congruity between the research methodology and the research question or objectives?	Yes	Yes	Yes	Yes	Unclear	Yes	Yes	Yes
Is there congruity between the research methodology and the methods used to collect data?	Yes	Yes	Yes	Yes	Yes	Yes	Yes	Yes
Is there congruity between the research methodology and the representation and analysis of data?	Yes	Yes	Yes	Yes	Yes	Yes	Yes	Yes
Is there congruity between the research methodology and the interpretation of results?	Yes	Yes	Yes	Yes	Yes	Yes	Yes	Yes
Is there a statement locating the researcher culturally or theoretically?	Yes	Unclear	Yes	No	Unclear	Unclear	Unclear	Unclear
Is the influence of the researcher on the research, and vice- versa, addressed?	Unclear	Unclear	Yes	No	Unclear	Unclear	Unclear	Unclear
Are participants, and their voices, adequately represented?	Yes	Yes	Yes	Yes	Yes	Yes	Yes	Yes
Is the research ethical according to current criteria or, for recent studies, and is there evidence of ethical approval by an appropriate body?	Yes	Yes	Yes	Yes	Yes	Yes	Yes	Yes
Do the conclusions drawn in the research report flow from the analysis, or interpretation, of the data?	Yes	Yes	Yes	Yes	Yes	Yes	Yes	Yes
**Overall appraisal**	Include	Include	Include	Include	Include	Include	Include	Include

#### Data extraction and synthesis

Data extracted from selected studies included standard information such as authors, setting, study findings and verbatim from participants reported (see Table 2). To synthesise the data, codes in the form of first and second order constructs were formulated from each primary study. These codes were organized into categories through a process of constant comparison across the studies [31]. This facilitated either translation of the studies into one another (areas of agreement across the studies) or refutational synthesis (areas of disagreement across the studies) based on the emerging categories. Following the translation process, the categories were re-interpreted to formulate overarching concepts/ metaphors. These overarching concepts/ metaphors formed the basis for undertaking a narrative synthesis [31]. The interpretive process was iterative with reference to the primary studies.

**Table 2:** Data extraction

Author/ year/ aim	Design/ methodology	Key findings	Sample quotes	Codes	Key/ overarching concepts/ metaphors
Sun et al., (2020) [32] To explore the psychological experiences of nurses caring for COVID-19 patients.	Descriptive phenomenology Purposeful sampling approach to recruit 20 nurses caring for patients with COVID-19 in Henan, China Face to face and telephone interviews conducted Data analysis by Colaizzi’s 7-step method Critical appraisal: Include	Four themes emerged: **• Significant number of negative emotions in the early stage:** this theme describes participants’ experiences with fatigue, discomfort and helplessness caused by the nature of caring for the patients and wearing the protective clothing. Also, there was fear of contracting the virus, anxiety caused by limited understanding of managing the infection/ presence of strangers as well as concerns about their family members	“After putting on protective clothing, nursing duties are awkward to carry out. Protective clothing needs to be worn for 8 h or more without drinking water and eating food and urinating was done with adult diapers.” “...The moment I walked through the door of the Department of Infectious Diseases, I felt very scared. I felt much better after I got used to it. And I felt scared when I pushed the door of the negative pressure room for the first time, but I was fine the second time.”	Initial psychological chaos; coping and thriving; support Sense of responsibility to care; psychological chaos; support1.1.1.1.	1. Surviving to thriving in an evolving space 2. Support amid the new normal1.1.1.1.
		**• Coping and self-care styles:** this theme describes the psychological/ life adjustment processes to face the situation and seeking support from one’s professional group.	“My method is not to think about stress, I shield it out of my life.” “...I forget everything when I am busy...” “We encourage each other. It does not feel like I’m fighting alone, I’m not afraid.”		
		**• Growth under pressure:** this theme describes the personal growth experienced by participants which include the opportunity for self-reflection, gaining a deeper sense of professional identity and increased affection and feelings of gratefulness.	“I used to work to earn a salary, but now it feels like a responsibility.” “Maybe there was a discrimination against nurses in the society but now I am proud of my choice.” “After work I find the sky is blue and everything is beautiful.” “I never thought I could be so strong.”		
		**• Positive emotions occurring simultaneously or progressively with negative emotions:** the theme describes the positive emotions that emerged in the process. These included feelings of confidence in the hospital environment, happiness from multiple sources of support and calmness during the care delivery process.	“I feel that the government has strong prevention and control measures, and the epidemic will be controlled very soon. But after all, we have a large population, and it is a process.” “Patients are very cooperative with our work. Although some patients have emotions due to illness, they show great respect to us.” “My mood is much better after starting pre-job training.” “Many colleagues called me to encourage me and I felt that there were many people who cared about me.”		
Liu et al., (2020) [33] To describe the experiences of nurses and doctors in the early stages of the COVID-19 outbreak	Descriptive phenomenology Purposive and snowball sampling was employed to recruit 9 nurses and 4 physicians in Hubei Province, China Data collection by in-depth telephone interviews Haase’s adaptation of Colaizzi’s method was used to analyse transcripts Critical appraisal: Include	Three themes with 10 subthemes emerged: **• Being fully responsible for patients’ wellbeing— “this is my duty”:** this theme describes a sense of duty expressed by participants. This included a feeling of being called to duty, caring for the affected persons, and emotionally supporting the patients.	“We must try our best to win this battle. As health-care providers, we are at the forefront. I fight for my family, and I fight more for this society.” “This is my duty because I am a medical worker. No matter what will happen” “Patients are struggling to breathe, and some can only lie in bed. They are very helpless and want care from their families.”		
		**• Challenges associated with working on the COVID-19 wards:** working in these wards was a completely new contexts with participants experiencing exhaustion and being overwhelmed with the workload, dealing with uncertainty and fear of contracting the virus/ infecting others, being witnesses of the patients’ experiences, and dealing with the healthcare provider-patient relationship amid the chaos.	“I was very tired. I had to lie in bed for a whole day to recover from the fatigue after work.” “I felt very depressed on the first day in the infectious disease hospital because there was only one entrance and passage for medical staff, and it is a real isolation unit with negative pressure. I felt it was difficult to breathe … This new environment brought a sense of oppression.” “I have to treat many patients who are not in my specialty. Although the country has released six editions of diagnosis and treatment guidelines [for COVID-19], there is still no effective antiviral medicine. It is an unknown disease, and everyone feels powerless.” “I recently contacted a colleague without any protection, who was later diagnosed with COVID-19. Although my CT results did not show any abnormality, I am anxious and waiting to do the throat swab.”		
		**• Many sources of social support to cope with the situation and transcendence:** This theme reflects participants’ sources of support including family, friends, colleagues, and the society.	“I am not overstrained because I trust our hospital. Our hospital gives us strong logistical support, including providing medical protective supplies, accommodations, transportation, food, medicines, and subsidies.” “The head nurse knows we come from different departments and infectious disease is not our specialty, so she sent us some educational videos and materials, and we can learn after work.” “When I feel stressful, I complain to my boyfriend. He is also a nurse, and we are in the same department. We communicate with and understand each other.”		
Kackin et al., (2020) [34] To determine the experiences and psychosocial problems of nurses caring for patients diagnosed with COVID-19 in Turkey.	Descriptive phenomenology Purposive sampling was employed to recruit 10 nurses caring for patients diagnosed with COVID-19 in Istanbul, Turkey Data collection was conducted via questionnaires and semi-structured interviews Colaizzi’s method was used to analyse transcripts Critical appraisal: Include	Three themes and ten subthemes emerged: **•** Effects of the outbreak: this theme highlights the working conditions, psychological and social effects of the outbreak. The nurses were faced with lack of equipment/ worsening work conditions, stress, feeling threatened, uncertain, depression, fear, aggression, social isolation (spending more time in the hospital) and stigma. **•** Short-term coping strategies: Participants emotions normalized (accepting the situation and thinking it is a temporal issue), refusal to dwell on the experiences, avoidance (avoiding the media/ comments about the disease), openly expressing their feelings (crying etc) and distraction (being thankful, listening to music, sports etc.) **•** Needs: nurses required ongoing psychological support and increasing resource availability at the setting.	“Nurses I have never known or seen. They were assigned to our service unit from another one. I don’t know their reactions. .. we had a dispute the other day with another Nurse. .. It feels as if working in another hospital. Different patients, a different order” “‘There is a patient lying there, you know that the patient needs you, but wearing that protective equipment, feeling his/her physical pain in your own body, you may have to work for an hour at most once you wear the helmet. It gives you a headache. You cannot enter the isolation rooms without those garments, and those garments are extremely smothering you. Sometimes, leaving the room when we admit new patients can take 2.5–3 h without exaggeration. When we leave, you find yourself in full of sweat “ “‘I left my family alone. .. My mother suffers from high blood pressure, what happens if she becomes infected. .. there is the fear of losing her.. ..”	Initial sense of psychological chaos; needing ongoing psychological support; coping	
Karimi et al., 2020 [35] To explore the lived experiences of nurses caring for patients with COVID-19 in Iran.	Descriptive phenomenology Purposive sampling was employed to recruit 12 nurses caring for patients diagnosed with COVID-19 in Iran Data collection was conducted via semi-structured interviews Colaizzi’s method was used to analyse transcripts Critical appraisal: Include	Three themes and six subthemes emerged: **•** Mental condition: The theme describes the psychological responses of healthcare providers which include stress, anxiety, and fear. These were related to fear of the disease, being worried about their families. **•** Emotional condition: the theme highlights feelings of suffering and affliction such as uncertainty about the disease process, witnessing death/ dying and separation from their own family **•** Care context: the theme highlights the turmoil and limited availability of support and equipment. Increasing work pressure, staff shortage, chaos and inexperience in handling the chaos	“Maybe I die, but I still have lots of dreams.” “I’m scared for my family and also for myself.” “How horrible these days are, we’re all dying.”	Initial psychological stress; navigating an unknown disease; dealing with resource limitations	
Galehdar et al., 2020 [36] To explore nurses’ experiences of psychological distress during care of patients with COVID-19.	Qualitative design Purposive sampling was employed to recruit 20 nurses caring for patients diagnosed with COVID-19 in Iran Data collection was conducted via semi-structured telephone interviews Conventional content analysis was used to analyse transcripts Critical appraisal: Include	Eleven categories and 5 subcategories emerged: **•** Death anxiety: the nurses experienced psychological distress witnessing the deaths of patients with feelings of helplessness when they could not do anything to alleviate the patient’s symptoms; concerns regarding the high mortality rates **•** Anxiety due to the nature/ severity of the illness, rate of spread and unknown dimensions of the disease	“It is agonizing to see a person deprived of breath, his heart failing, and you can’t do anything about his suffering .... it sometimes causes me to feel agitated and distressed and becoming really sad and confused about what I’m going to do?” “I myself was caring for a patient with COVID-19, it was really painful to see a person striving to breathe to save himself “ “In my opinion, the nature of the disease is beyond what we are teaching and learning now”	Psychological issues and navigating the death of patients	
Ardebili et al., 2020 [37] To undertake an in-depth exploration of the experiences of health-care staff working during the COVID-19 crisis.	Qualitative design Purposive sampling was employed to recruit 97 healthcare professionals (pre-hospital emergency services (EMS), physicians, nurses, pharmacists, laboratory personnel, radiology technicians, hospital managers and managers in the ministry of health who work directly or indirectly with COVID-19 cases) caring for patients diagnosed with COVID-19 in Iran Data collection was conducted via semi-structured interviews Thematic analysis was used to analyse transcripts Critical appraisal: Include	Three themes and eleven subthemes emerged: **•** Working in the pandemic era: This was experienced as high workload and feelings of losing control over the situation, fear, anxiety, and being overwhelmed (Providing futile care) **•** Changes in personal life and enhanced negative effects **•** Gaining experience, normalization and adapting to the pandemic (overcoming the initial crisis, gaining experience regarding patient management, reducing referrals and increasing recoveries). **•** Mental health issues: Experiences of loss of control, heavy workload, severe stress, the experience of a sense of futile care, fear of infection and transmission, self-isolation, and quarantine, decreased emotional relationships, fundamental changes in lifestyle, worrying about the future and the economic situation, all appeared to contribute towards the manifestation of mental health issues).	*“In the early days, our workload was very high, we had to move the wards and hospitalized corona patients in the non-infectious wards”* “*Every day a new drug is introduced, every day a new route of transmission is introduced”* *“This disease does not have a specific drug, nor can you predict with confidence who will survive and who will die. This made me feel (completely ineffectual and I felt) like I was losing control”* “*It’s very difficult to wear N95 masks for 12 h, I feel short of breath and I will definitely have problems later (Nurse)* *They give (you) a body suit in each shift. When we wear these clothes, sweat flows from all over our bodies, we can’t eat anything with these clothes, we can’t drink anything too, we have to wear them for 12 h”* “*When I was hospitalized in the ICU, I had very severe shortness of breath. When the shortness of breath was present, I thought I was dying (Nurse) I was thinking, I will die alone, without seeing my family, they will not see my body. I will not have a proper funeral”*	Initial psychological distress; facing personal changes; adjusting to the situation	
Al Ghafri et al., 2020 [38] To explore the experiences and perceptions of health care workers (HCWs) in primary health care in the management of COVID-19 with respect to medical response experiences, socio-cultural and religious reforms, psychological impressions, and lessons learned.	Phenomenology Purposive and snowball sampling was employed to recruit 40 healthcare professionals/ stakeholders involved in managing patients diagnosed with COVID-19 in Oman Data collection was conducted via focus group discussions (6 focus group discussions conducted) Thematic analysis Critical appraisal: Include	Three themes emerged: **•** Medical response experiences: rapid restructuring of public health services, enforcing technology use and increasing burden on limited human resource available. **•** Socio-cultural and religious reforms: having to stay away from parents and families (inability to participate in social/ religious practices); empathy towards the vulnerable in the society **•** Psychological issues: being at home was described as depressing, and inability to travel around were distressing; exhaustion among healthcare professionals and fear of transmitting the virus to families/ loved ones.	“we had to work for more than 12 h continuously due to shortages of staff. This was an overburden to us” ““when our colleagues got infected, we all suffered physically and emotionally”	Psychological concerns; adjusting the healthcare system	
Nyashanu et al., 2020 [39] To explore the triggers of mental health problems among frontline healthcare workers during the COVID-19 pandemic.	Exploratory qualitative study Purposive sampling was employed to recruit 40 frontline staff involved in in private care homes and domiciliary care agencies in the Midlands, UK Data collection was conducted via semi-structured interviews Interpretive phenomenological analysis Critical appraisal: Include	Seven themes noted in the study: **•** Fear of infection and infecting others **•** Lack of recognition **•** Lack of guidance/ frequently changing guidelines creating doubts about operational procedures and triggering anxiety **•** Unsafe hospital discharge **•** Loss of professionals/ residents through death **•** Unreliable testing and delayed results **•** Staff shortage causing anxiety and worry	“Unfortunately, there have been so many changes on the guidance to COVID-19. Being diabetic the government has placed responsibility on my employer to make suitable safe working arrangements which is difficult. A female learning disability nurse I am really worried with ever changing information from government on how to act during this pandemic …. Honestly, it really makes me anxious” “We have been using agency staff to maintain staff numbers, but we don’t know where else they have been working and this brings so much anxiety.”	Fear, anxiety, worry; lack of guidelines; limited human resources	

#### Findings

##### Study characteristics

Following the screening process, eight primary qualitative studies met the criteria for inclusion in this review [32–39]. Although all the studies involved healthcare staff caring for patients with COVID-19, majority focused on nurses (see Table 1). The settings of the primary studies include Mainland China [32, 33], Iran [35–37], Turkey [34], Oman [38] and United Kingdom [39]. Five studies utilised a phenomenological approach to uncover participants’ lived experiences [32–35, 38]. All studies received an overall appraisal as “include”.

##### Concepts/ metaphors

The interpretation of the data and translation of the studies into each other led to the emergence of two overarching concepts/ metaphors: surviving to thriving in an evolving space and support amid the new normal (see Table 3). The relationship between the emerging concepts/ metaphors was noted to be reciprocal which facilitated the development of a line of argument to understand the phenomenon of caring for patients with COVID-19.

**Table 3:** Metaphors/ concepts and codes

Metaphors	Codes
Surviving to thriving in an evolving space	• Initial psychological/ emotional chaos
	• Living and functioning in a ‘new body’
	• *Thriving amidst chaos*
Support amid the ‘new normal’	• Support systems
	• Clinical guidelines

#### Surviving to thriving in an evolving space

##### Initial psychological/ emotional chaos

The COVID-19 emerged as an infection to which healthcare staff initially had limited knowledge. Thus, being asked to work on a ward for persons with the infection created an initial sense of inner tension/ psychological chaos and an ‘internal’ struggle to survive in an uncertain dimension of a rapidly evolving disease [32–39]. Psychological responses such as anxiety, helplessness, fear of contracting the infection and spreading to one’s loved ones, and uncertainty characterised the initial survival space and trickled to the thriving phase [32–39]:

*“Although I volunteered to work in the Department of Infectious Diseases, I still feel very scared. After all, it is a new infectious disease and there are no specific drugs at present. I was scared to see reports of the sacrifice of medical staff in other cities.”* [32]

*“ … we are fearful of being infected. Anyone who coughs in the office causes panic. If one is infected, all medics in the unit are in danger, then the unit will be paralysed... I recently contacted a colleague without any protection, who was later diagnosed with COVID-19. Although my CT results did not show any abnormality, I am anxious and waiting to do the throat swab.”* [33]

*“I’m not calm at all, and I do not know what’s going on”* [35]

The fears of some healthcare staff came to fruition as they contracted the COVID-19 disease. This led to feelings of social isolation as they received treatment and hanging in a balance as they navigated through the symptoms on their own. Within the space of contracting the disease, affected healthcare staff were faced with new fears regarding dying alone with their mortal remains not receiving the final respect required [34–38].

“*When I was hospitalized in the ICU, I had very severe shortness of breath. When the shortness of breath was present, I thought I was dying (Nurse) I was thinking, I will die alone, without seeing my family, they will not see my body. I will not have a proper funeral”* [37]

The initial psychological chaos experienced by the healthcare staff heightened as they witnessed varying mortality rates [32, 33, 35–37]. The most challenging aspect for healthcare staff appeared to be contracting the illness themselves or witnessing the death of a colleague following a diagnosis of COVID-19 [37–39]:

*“When our colleagues got infected, we all suffered physically and emotionally”* [38]

*“It is agonizing to see a person deprived of breath, his heart failing, and you can’t do anything about his suffering .... it sometimes causes me to feel agitated and distressed and becoming really sad and confused about what I’m going to do?”* [36]

##### Living and functioning in a ‘new body’

The survival phase was also characterised by struggling to live and function in a ‘*new body*’, that is the personal protective equipment (PPE) which appeared to be uncomfortable, yet indispensable [32, 33, 37, 38]. These concerns notwithstanding, healthcare professionals felt a sense of responsibility to fight the illness, care for the persons diagnosed with COVID-19, protect themselves and their loved ones from contracting the “deadly virus” [32–39]:

*“After putting on protective clothing, nursing duties are awkward to carry out. Protective clothing needs to be worn for 8 hours or more without drinking water and eating food and urinating was done with adult diapers.”* [32]

*“Wearing the whole set of PPEs is very uncomfortable. I have difficulty breathing and feel very hot and my heart rate speeds up. We keep on sweating and the clothes are soaked.”* [33]

“*It's very difficult to wear N95 masks for twelve hours, I feel short of breath and I will definitely have problems later”* [37]

*“We must try our best to win this battle. As health-care providers, we are at the forefront. I fight for my family, and I fight more for this society. This is my duty because I am a medical worker. No matter what will happen”* [38]

##### Thriving amidst chaos

As healthcare staff continued to navigate through the evolving space of care provision, received training, and identified strategies of survival, there was a gradual move from survival to thriving which was characterised by resilience [32–38]. In the thriving phase, participants did not still understand the nature of the infection fully but felt more at ease working with the affected persons. The thriving phase was also characterised by personal and professional growth in the face of adversity with a feeling of being in a supportive environment, although resources were still limited [32–38]. Further within the thriving phase, healthcare staff began to appraise the negative experiences in a positive manner as a means of coping within a context that was not fully understood [32–38]. Irrespective of the phase healthcare staff found themselves, they were still faced with fear, increasing workload which led to exhaustion as they navigated through patient care and their own experiences [32–39]:

*“My method is not to think about stress, I shield it out of my life...I forget everything when I am busy...”* [38]

*“In the early days, our workload was very high, we had to move the wards and hospitalized corona patients in the non-infectious wards”* [37]

#### Support amid the ‘new normal’

##### Support systems

The need for ongoing psychological support to help manage the ‘self’ was highlighted by all studies as healthcare staff navigated through survival to thriving [32–38]. Support from other members of the healthcare team was considered essential as the disease was considered a common ‘enemy’ among staff [32–38]. Beyond the confines of the hospital, some healthcare staff also received support from families and friends [32, 33]. Aside managing the ‘*self’*, healthcare staff also required support in utilizing the limited resources, ongoing training to stay updated about the disease and how best to protect oneself and family [32–38]:

*“When I feel stressful, I complain to my boyfriend. He is also a nurse, and we are in the same department. We communicate with and understand each other.”* [35]

*“The head nurse knows we come from different departments and infectious disease is not our specialty, so she sent us some educational videos and materials, and we can learn after work.”* [33]

##### Clinical guidelines

Rapidly changing guidelines were challenging for healthcare staff and they required more support regarding operational procedures [39]:

*“Unfortunately, there have been so many changes on the guidance to COVID-19. Being diabetic the government has placed responsibility on my employer to make suitable safe working arrangements which is difficult. A female learning disability nurse I am really worried with ever changing information from government on how to act during this pandemic … . Honestly, it really makes me anxious”* [39]*.*

## Discussion

The review sought to gain a comparative understanding of the experiences of healthcare staff in caring for persons with the novel COVID-19 disease. The findings highlight an initial sense of psychological chaos with healthcare staff struggling to survive as they navigated through the outbreak. Overtime, healthcare staff transitioned from survival to thriving as they continued to provide care but still experienced heavy workload, emotional exhaustion, and fear of contracting the disease or transmitting to family members/ loved ones. Some healthcare staff who contracted the disease also experienced fear of dying alone. Besides, though healthcare staff may experience growth under pressure, the presence of heavy workload and emotional exhaustion may highlight the potential of burnout, secondary traumatic stress, and subsequently, compassion fatigue. The impact of these psychological experiences emphasises the need for early and ongoing psychosocial support as well as maintaining high standards of infection prevention and control measures to make healthcare staff feel safe. Continuing professional education on emerging trends of the disease, ensuring the availability and utilisation of safety materials, promoting team morale, and providing avenues of release for healthcare professionals are also needed to support staff caring for persons with COVID-19.

The initial phase of working with persons infected with the novel virus is a critical period of transitioning to an unknown context with varied emotional responses heightening around a week of entering this unfamiliar space [19, 32–39]. In a previous study among Korean nurses during the era of the Middle East Respiratory Syndrome (MERS), the unfamiliar space of the infection was described as a dangerous field filled with psychological and physical stressors [40]. Additionally quantitative studies that evaluated the presence of mental health issues during the ongoing pandemic have underscored the presence of high levels of anxiety, depression, and fear among healthcare staff [41–45]. As previously mentioned, healthcare staff who work with critically ill persons already face several stressors which predispose them to burnout and compassion fatigue [46, 47]. Extrapolating these stressors and experiences to the context of an unknown illness suggest the existence of a significant psychological and emotional challenge among healthcare staff caring for persons with COVID-19. It is worth mentioning that though healthcare staff may experience personal and professional growth under pressure, they are still faced with significant workload levels and emotional exhaustion. Thus, the risk of burnout, traumatic stress, and compassion fatigue are still present. Without professional support, healthcare staff are at a risk of several issues such as insomnia, mood and eating disorders, in both short and long term [1, 48]. These findings should therefore direct our attention towards early mental health intervention to identify, acknowledge and offer support commensurate to the needs of healthcare staff [19]. For instance, a brief onsite mindfulness-based intervention has been reported to be feasible, safe, and potentially helpful in supporting frontline workers [49]. Other cognitive-based therapies need to be evaluated to ascertain their impact in improving outcomes [50].

Further to the above, fears about contracting the COVID-19 disease and/ or infecting one’s family members/ loved ones emerged as a major source of stress among healthcare staff. Even when the initial experience of psychological chaos was low with existing high standards of infection prevention strategies, fear of contracting the disease remained a significant concern among healthcare staff [51–57]. Healthcare staff who contracted the disease were faced with additional fears of dying alone with feelings of being socially isolated from colleagues and family/ loved ones as they underwent treatment. The findings strengthen the need for policies to make clinicians well-being a priority across healthcare settings and countries [58]. Healthcare staff need to feel safe within the healthcare setting whilst ensuring adherence to best infection prevention and control practices [15]. Avenues for healthcare staff to express their fears are needed to help them navigate through their emotions [59]. Additionally, healthcare staff who contract the illness may require extra support to deal with emerging psychological/ emotional impact of the illness.

Despite the emergence of several stressors, some facilitators to surviving/ thriving emerged. Key among these facilitators is the support offered by peers within one’s team and family. Previous studies have highlighted the significant role played by peers at the workplace as there seem to be a shared concern among these persons who are journeying together within unfamiliar territories [40, 60]. In fact, lack of social support has been linked to the development of anxiety, insomnia, and depression [57, 61, 62]. Peer support develops overtime and creates a sense of connectedness which may be difficult to quantify. This form of unique support requires further attention to determine ways of facilitating their development and improvement particularly in this era of journeying through a common ground.

The review findings offer insights into the experiences of healthcare professionals caring for persons with COVID-19. A notable strength is the translation of the primary studies into each other to generate a comparative understanding of the phenomenon of caring for persons with COVID-19. Some limitations are however noteworthy. Majority of the participants in the primary studies were nurses which creates the need to engage other healthcare staff such as laboratory technicians and mortuary attendants to understand their experiences. Additionally, although the review findings facilitated a reciprocal interpretation, the findings may not necessarily apply to other settings. Besides, generalizing the findings to the wider healthcare population may not be possible. It is also worth mentioning that only studies reported in English were included in this review.

## Conclusion

Navigating through the experiences of healthcare staff during the outbreak highlight the existence of several concerns warranting attention. Although professional/ personal growth may be experienced, healthcare staff are still faced with heavy workload and emotional exhaustion which can predispose them to burnout and compassion fatigue. The findings have significant policy and practice implications such as a need for early and ongoing psychosocial support, support in handling fears, ensuring the availability of required equipment and identify strategies to boost team morale. As noted in a recent editorial, a period of rebuilding, resetting, and recovery is needed placing frontline healthcare staff at the front and centre of recovery measures [63].

## Data Availability

The datasets used and/ or analyzed during the current study are included in this published article.
